# The Role of TNPO3 in HIV-1 Replication

**DOI:** 10.1155/2012/868597

**Published:** 2012-07-19

**Authors:** Felipe Diaz-Griffero

**Affiliations:** Department of Microbiology and Immunology, Albert Einstein College of Medicine, 1301 Morris Park, Price Center 501, New York, NY 10461, USA

## Abstract

TNPO3, transportin-SR2 or Tnp3, a member of the karyopherin **β** superfamily of proteins, is important for the ability of human immunodeficiency virus (HIV-1) to achieve productive infection, as TNPO3 depletion in human cells leads to a dramatic reduction of infection. Here we describe and discuss recent findings suggesting that TNPO3 assists HIV-1 replication in the nucleus and in fact that TNPO3 may assist PIC maturation in the nucleus. In addition, the viral determinant for the requirement of TNPO3 in HIV-1 infection is discussed. This paper summarizes the most significant recent discoveries about this important host factor and its role in HIV-1 replication.

## 1. Introduction

The influence of the physiological state of cells on retroviral replication has been known since Temin and Rubin demonstrated that stopping cell division by X-rays or UV light prevents Rous sarcoma virus replication [1]. Subsequent research established the relationship between cell cycle stage and retroviral infection, revealing that retroviruses do not all have the same requirements for productive infection [2, 3]. For example, *γ*-retroviruses such as murine leukemia virus (MLV) require the host cell to pass through mitosis for efficient infection [4, 5]. The MLV titer decreases at least 10-fold when infecting cells that are arrested in a non-dividing state. By contrast, lentiviruses such as HIV-1 show no difference in productive infection in dividing versus nondividing cells [6]. This evidence suggests that lentiviruses have developed specific mechanisms for the infection of non-dividing cells. The ability of HIV-1 to infect non-dividing cells has been attributed to its capacity to transport the preintegration complex (PIC) to the nucleus [7, 8]. Translocation of the HIV-1 PIC into the nucleus is not a simple process as the PIC is a large complex that contains integrase, matrix, capsid, Vpr, and the viral DNA [7, 9, 10]. Because of its large size, it is unlikely that the PIC enters the nucleus by passive diffusion [11]. On the contrary, HIV-1 PIC translocation into the nucleus must be an active process, possibly making use of nuclear localization signals [12]. Several viral components of the PIC such as matrix, Vpr, integrase, and the central DNA flap have been proposed to be directly involved in PIC transport into the nucleus. However, evidence in the literature both supports and refutes a role for these different components in nuclear translocation [13, 14]. Although only small amounts of capsid can be found in biochemically purified HIV-1 PICs [7, 12, 15, 16], evidence has shown that capsid plays an important role in the ability of HIV-1 to infect non-dividing cells [3, 17–19] The mechanism used by the HIV-1 PIC to enter the nucleus is not completely understood; however, it is widely accepted that nuclear import of the complex is active and energy dependent [8].

In addition to the viral determinants involved in HIV-1 PIC nuclear import, several host factors have been implicated in the process: (1) importin 7 [20–22], (2) importin *α*3 [23], (3) importin/importin heterodimer [20, 24, 25], (4) NUP153 [19, 26, 27], (5) RanBP2 [28], and (6) TNPO3/transportin-SR2 [29–35]. 

TNPO3, transportin-SR2 or Tnp3, a member of the karyopherin *β*  superfamily of proteins, is important for the ability of HIV-1 to achieve productive infection, as TNPO3 depletion leads to a reduction of HIV-1 infectivity [29–37]. TNPO3 transports pre-mRNA splicing factors into the nucleus [38] and recognizes them by binding to phosphorylated or nonphosphorylated serine/arginine-rich motifs in a RanGTP-dependent manner [39, 40]. TNPO3 is also an export factor for certain tRNA species, and its yeast ortholog Mtr10p is an export factor for small ribosomal subunits [36, 41]. 

## 2. Role of TNPO3 in Retroviral Infection

The role of TNPO3 in retroviral infection was initially discovered for HIV-1 [30]; however, more recent work has demonstrated that TNPO3 is also important for infection by HIV-2, simian lentiviruses, and, to a lesser extent, equine infectious anemia virus (EIAV) [31, 32, 37, 42] but not MLV or Feline immunodeficiency virus (FIV). Intriguingly, simian immunodeficiency viruses (SIVs) exhibited the strongest dependency on TNPO3 for infection [31, 32, 37, 42]. 

## 3. Viral Determinants for the Requirement of TNPO3

### 3.1. Integrase

**Figure 1:**
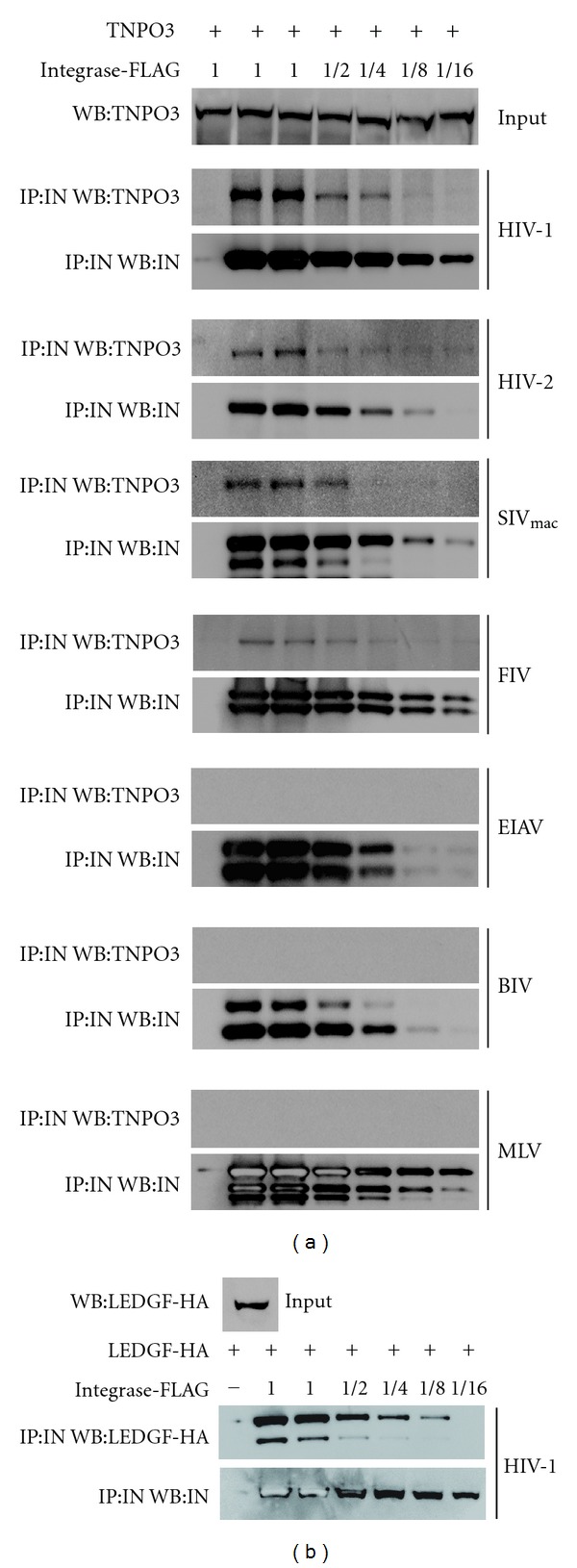
TNPO3 Interaction with retroviral integrases. (a) Human 293T cells, which endogenously express TNPO3, were transfected with different amounts of the indicated mammalian codon-optimized FLAG-tagged retroviral integrases (IN). Twenty-four hours following transfection cells were lysed in extraction buffer (400 mM NaCl, 0.5% Triton X-100, 50 mM Tris-HCl, pH = 8, 2 mM MgCl^2^, 5% glycerol and protease inhibitors (Roche)). Subsequently, extracts were treated with DNAase and precleared using protein-A agarose beads (Sigma) at 4°C for 1h. Small aliquot of the initial extract was analyzed by Western blot (WB) using anti-TNPO3 antibodies (INPUT). Subsequently, the extracts were used to immunoprecipitate (IP) the different retroviral integrases using anti-FLAG antibodies. FLAG-peptide eluted complexes were analyzed by WB for the presence of TNPO3 and using anti-TNPO3 and anti-FLAG antibodies, respectively. (b) As a positive control we assayed the known ability of HIV-1 integrase to interact with LEDGF/p75. For this purpose, HA-tagged LEDGF/p75 (LEDGF-HA) was cotransfected together with FLAG-tagged HIV-1 integrase and immunoprecipitated using anti-FLAG beads. Eluted complexes were analyzed for the presence of LEDGF/p75 and HIV-1 integrase by WB using anti-HA and anti-FLAG antibodies, respectively. Similar results were obtained in three independent experiments, and the results of a representative experiment are shown.

**Figure 2:**
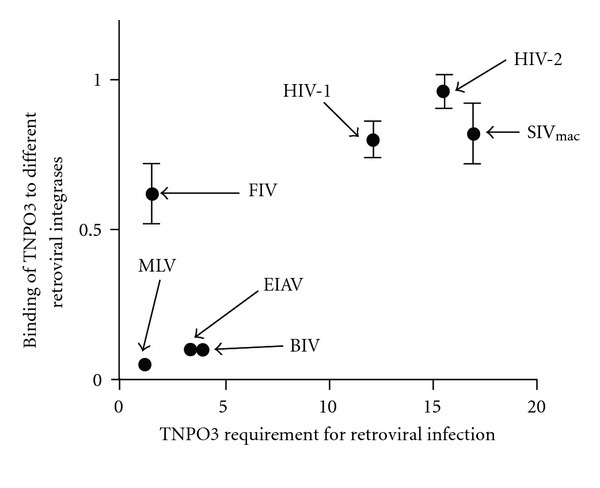
Correlation of the ability of TNPO3 to bind different retroviral integrases with the requirement of TNPO3 in retroviral infection. The ability of TNPO3 to bind to different retroviral integrases was calculated by quantifying the amount of bound TNPO3 relative to the amount of immunoprecipitated integrase specified in Figure 1. The requirement of TNPO3 for the indicated retrovirus was calculated by the fold inhibition in TNPO3-depleted cells when 50% of wild-type cells were infected.

A yeast two-hybrid screen identified TNPO3/transportin SR-2 as a host protein that interacts with HIV-1 integrase [29]. These studies confirmed that TNPO3 does, indeed, bind to integrase, suggesting that integrase may be a key viral determinant for the requirement of TNPO3 in productive HIV-1 infection; the same work showed that endogenously expressed TNPO3 in mammalian extracts binds recombinant HIV-1 but not MLV integrase, which agrees with the result that TNPO3 is required for HIV-1 infection but not for MLV [29]. By contrast, the use of recombinant integrases from different retroviruses demonstrated that bacterially purified GST-TNPO3 binds to integrase proteins of HIV-1, MLV, SIVmac, FIV, bovine immunodeficiency virus (BIV), and with less affinity to the integrase of EIAV [31]; this latter result fails to correlate TNPO3 binding to integrase with the requirement for infectivity. We also tested this correlation by using both TNPO3 and viral integrases from mammalian extracts. By pulling-down codon-optimized integrases from different retroviruses expressed in mammalian cells, we demonstrated that endogenous TNPO3 binds HIV-1, HIV-2, and SIVmac integrases, which correlates with the requirement for TNPO3 on infectivity (Figure 1(a)). Similarly, we observed that the FIV integrase binds TNPO3, though somewhat weakly. In contrast, the integrase proteins of EIAV, BIV, and MLV did not bind TNPO3 in this particular assay (Figure 1(a)). As a positive control for binding, we demonstrated that, under similar pull-down conditions, lens epithelium-derived growth factor (LEDGF)/p75 bound HIV-1 integrase (Figure 1(b)). Interestingly, we found a positive correlation between TNPO3 binding and the requirement for TNPO3 in primate lentiviral infection (Figure 2). Although western blot is a semiquantitative assay, it provides a trend. Overall, in the case of lentiviruses, a correlation exists between TNPO3 binding to integrase and the requirement of TNPO3 in infection The fact that the integrase of FIV interacts with TNPO3 and that TNPO3 is not required for FIV infection suggests the existence of two distinct groups of viruses. However, there is no genetic evidence pointing to integrase as the determinant for the requirement of TNPO3 during infection. Indeed, generation of such evidence might not be an easy task, given that integrase mutants affect multiple stages of the viral life cycle and complicate clear interpretation of phenotypes [43].

The differences observed in the interaction of TNPO3 and viral integrases could lie on the origin of the protein used to measure the interactions. However, it is difficult to determine which approach is closer to the interactions that occur inside the cell. In conclusion, TNPO3 binds integrase; however, the role of this interaction during retroviral infection is not understood.

### 3.2. Capsid

In contrast to integrase, genetic and biochemical evidence exists for capsid as a determinant for the requirement of TNPO3 during HIV-1 infection [31, 36, 37, 44]. By using HIV/MLV chimera viruses on the capsid protein, the Engelman Lab demonstrated that capsid is the genetic determinant for the requirement of TNPO3 during infection [31]. Similarly, by extensive mutagenesis of capsid, the Luban Lab demonstrated that capsid plays a major role in the requirement for TNPO3 during infection [44]. TNPO3 was reported to bind soluble capsid [36], and, more recently, a direct biochemical interaction between TNPO3 and the HIV-1 core has been demonstrated in our laboratory [37]. Interestingly, we found that TNPO3 binds to HIV-1 capsid-nucleocapsid complexes that have been assembled *in vitro*, which recapitulate the surface of the viral core [45]. Altogether, this evidence points out capsid as an important determinant for the requirement of TNPO3 during productive HIV-1 infection.

## 4. Role of TNPO3 in HIV-1 Nuclear Import

It is believed that TNPO3 is involved in nuclear import of the HIV-1 PIC on the basis of the following evidence [29]: (1) reduction in the number of 2-LTR circles during HIV-1 infection of TNPO3-depleted cells when compared to infection of wild-type cells and (2) observation of decreased nuclear translocation of the PIC in TNPO3-depleted cells by using an HIV-1 virus containing an IN-GFP fusion protein. It should be noted, however, that this interpretation is in question, as more recent work has detected no change in HIV-1 nuclear entry in the face of TNPO3 depletion, implying that the block is subsequent to nuclear import [34, 36, 37, 42, 44]; the different groups who have investigated this issue demonstrated that the number of HIV-1 2-LTR circles in TNPO3-depleted cells was similar when compared to wild-type cells.

It is important to mention that the measurement of 2-LTR circles is indirect evidence of PIC nuclear import. After the viral DNA is imported into the nucleus, it integrates into the genome; however, a fraction of this viral DNA is ligated to produce circular forms by nuclear DNA ligases [46]. These products are known as 2-LTR circles, and they are used as indirect measure of nuclear import. Although the 2-LTR is an indirect measure of PIC nuclear import, this methodology is widely used as a marker of nuclear import [46].

 Furthermore, no difference was observed in the levels of viral DNA nuclear accumulation in TNPO3-depleted cells relative to control cells following biochemical fractionation, which supports the 2-LTR findings [36]. Altogether, the work from several independent laboratories suggests that TNPO3 is required when the PIC is in the nucleus.

## 5. Role of TNPO3 in Nuclear Maturation of the PIC

The consensus that TNPO3 assists HIV-1 replication in the nucleus led to testing of the hypothesis that TNPO3 may be promoting a nuclear maturation step [36]. Remarkably, the Fassati group demonstrated that more capsid accumulates in the nucleus of TNPO3-depleted cells during HIV-1 infection relative to wild-type cells. These results indicate that the presence of TNPO3 in wild-type cells contributes to the removal of capsid from the nucleus, which may be important for PIC maturation in the nucleus and integration. In agreement with a role of TNPO3 in the nucleus, depletion of TNPO3 altered the selection of chromosomal sites for viral integration [28].

## 6. Role of CPSF6 in the Ability of TNPO3 to Assist HIV-1 Replication

The cleavage and polyadenylation specificity factor subunit 6 (CPSF6), an SR-protein, is a potential nuclear transport cargo of TNPO3. Interestingly, expression of a CPSF6 fragment (1-358) lacking the nuclear localization signal blocks HIV-1 nuclear import [19]; therefore, it is conceivable that TNPO3 depletion causes accumulation of CPSF6 in the cytosol and that this accumulation impairs HIV-1 replication. Furthermore, a virus containing the capsid mutation N74D is resistant to the replication block imposed by overexpression of the CPSF6 fragment in the cytosol [19]. Intriguingly, infection of the HIV-1 capsid mutant N74D is independent of TNPO3. Altogether, these results imply that the effect of TNPO3-depletion on HIV-1 infection is in part linked to CPSF6. 

Analysis of TNPO3-depleted cells revealed minimal changes in the distribution of CPSF6 by cytosolic/nuclear fractionation and immunofluorescence in HeLa cells [37]. These results implied that TNPO3 depletion minimally changes the localization of CPSF6 and suggest that the effect of TNPO3 depletion on HIV-1 infection is independent of a change in CPSF6 localization. However, these results do not exclude the possibility that CPSF6 plays a role in the phenotype observed for HIV-1 in TNPO3-depleted cells. 

## 7. Role of TNPO3 in HIV-1 Infection

TNPO3 is a nuclear importer that is important for HIV-1 replication. Two possible viral determinants of the requirement for TNPO3 in HIV-1 replication have been postulated, integrase and capsid [29, 31, 37, 44]. However, compelling genetic and biochemical evidence has only been found for capsid [31, 37, 44], and thus it remains a question whether integrase is still a player in the ability of TNPO3 to assist HIV-1 replication once infection has taken place. It is possible that capsid and integrase are jointly playing a role in the requirement for TNPO3 in HIV-1 replication. However, this remains to be determined.

Several groups have confirmed the observation that TNPO3-depletion allows formation of 2-LTR circles during HIV-1 infection [36, 37, 42, 44]. Even though formation of 2-LTR circles is an indirect measure of nuclear import, it is one of the most used tools to determine whether the HIV-1 PIC has been transported to the nucleus [46]. These experiments implied that in TNPO3-depleted cells the PIC has been transported to the nucleus; however, HIV-1 integration did not occur [29, 34, 36, 37, 44]. This suggested, in turn, that TNPO3 is assisting some process in the nucleus prior to integration. In agreement with this idea, it has been proposed that TNPO3 plays a role in depleting capsid from the nucleus during infection, which may help PIC maturation in the nucleus [36]. This model suggests that small amounts of HIV-1 capsid that remain bound to the PIC are transported into the nucleus, in agreement with the observation that capsid is the viral determinant for the infection of nondividing cells [17, 18]. However, experiments indicate that biochemically purified PICs contain very little capsid [7, 12, 15, 16], and in fact the presence of capsid in the nucleus during HIV-1 infection has not been clearly established. Future work should attempt to clarify this exciting possibility.

 An alternative hypothesis is that TNPO3 binding to the HIV-1 core in the cytoplasm aids the ribonucleoprotein (RNP) complex in a process required only after the complex enters the nucleus [37]. TNPO3 binding to the HIV-1 core may assist the maturation of the PIC in the cytosol; however, assistance provided by TNPO3 to HIV-1 replication in the cytosol will only be noticed when the complex reaches the nucleus. For example, 3′ processing activity of integrase on the HIV long terminal repeats (LTRs) has been suggested to occur in the cytoplasm [7, 47]. It is possible that TNPO3 binding to the HIV-1 core ensures proper 3′-processing of the viral LTRs in the cytoplasm, which will be important for viral integration when the complex reaches the nucleus. Future experiments should test whether TNPO3 depletion can affect 3′-processing of viral LTRs.

The discovery of TNPO3 has pushed the HIV-1 research community to explore in greater depth the mechanism by which HIV-1 crosses the nuclear envelope and integrates into the cellular genome. It is expected that this field will grow steadily in the coming years and bring to light novel mechanistic information and therapeutic opportunities.
